# Role of Testosterone Signaling in Microglia: A Potential Role for Sex‐Related Differences in Alzheimer's Disease

**DOI:** 10.1002/advs.202413375

**Published:** 2025-03-24

**Authors:** Haiyan Du, Akiko Mizokami, Junjun Ni, Simeng Zhang, Yosuke Yamawaki, Tomomi Sano, Eijiro Jimi, Isei Tanida, Takashi Kanematsu

**Affiliations:** ^1^ Department of Cell Biology, Aging Science, and Pharmacology Division of Oral Biological Sciences Faculty of Dental Science Kyushu University 3‐1‐1 Maidashi, Higashi‐ku Fukuoka 812‐8582 Japan; ^2^ OBT Research Center Faculty of Dental Science Kyushu University 3‐1‐1 Maidashi, Higashi‐ku Fukuoka 812‐8582 Japan; ^3^ Key Laboratory of Molecular Medicine and Biotherapy Department of Biology School of Life Science Beijing Institute of Technology Beijing 100081 China; ^4^ Department of Advanced Pharmacology Daiichi University of Pharmacy 22‐1 Tamagawa‐cho, Minami‐ku Fukuoka 815‐8511 Japan; ^5^ Laboratory of Molecular and Cellular Biochemistry Division of Oral Biological Sciences Kyushu University 3‐1‐1 Maidashi, Higashi‐ku Fukuoka 812‐8582 Japan; ^6^ Department of Cellular and Molecular Neuropathology Juntendo University Graduate School of Medicine Tokyo 113‐8421 Japan

**Keywords:** Alzheimer's disease, autophagy, microglia, sex‐related differences, testosterone

## Abstract

Alzheimer's disease (AD) is less prevalent in men than in women, although mechanisms remain unclear. Microglia degrade aggregated amyloid β (Aβ) through the lysosomal system, including autophagy. G protein‐coupled receptor family C group 6 member A (GPRC6A), predominantly expressed in mouse microglial MG6 cells, is a primary mediator of testosterone signaling. This study examines testosterone's role in modulating Aβ‐induced autophagy in microglia. Testosterone promotes Aβ‐induced autophagy leading to Aβ clearance in MG6 cells by suppressing extracellular signal‐regulated kinase (ERK) phosphorylation and subsequently inhibiting mammalian target of rapamycin (mTOR) activation, which is abrogated by shRNA knockdown of GPRC6A. In in vivo experiments with male 5xFAD AD model mice, Aβ clearance activity is associated with autophagy in microglia and is reduced by orchiectomy, but restored by testosterone supplementation. ERK phosphorylation in the brains of male AD model mice is upregulated by orchiectomy. Therefore, testosterone is involved in autophagy‐mediated Aβ clearance in microglia. Aβ accumulation in human brain samples from patients with AD is significantly lower in men than in women, with less pronounced colocalization of Aβ with p62 aggregates, suggesting enhanced autophagic activity in men. In conclusion, testosterone enhances Aβ‐induced autophagy in microglia, possibly contributing to lower susceptibility to AD in men.

## Graphical abstract

Testosterone enhances Aβ‐induced autophagy in microglia via the non‐genomic testosterone receptor GPRC6A, promoting Aβ clearance by suppressing ERK phosphorylation and inhibiting mTOR activation. In male 5xFAD mice, testicular removal reduces microglial autophagy and Aβ clearance, which is restored by testosterone. Human AD brains show lower Aβ accumulation in men, suggesting testosterone‐driven autophagy may contribute to reduced AD susceptibility. This figure was created with Biorender.com.

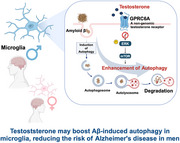

## 1 Introduction

Alzheimer's disease (AD) is a neurodegenerative disease characterized by multiple pathological features that collectively contribute to progressive cognitive decline. The hallmark of AD from its early onset is the extracellular accumulation of amyloid β (Aβ) plaques and intracellular neurofibrillary tangles formed by hyperphosphorylated tau proteins.^[^ ^1^, ^2^ ^]^ These pathological features contribute to a widespread impact on brain function, with notable differences observed between men and women. Sex‐based differences in AD prevalence and severity are well documented. Approximately two‐thirds of patients with AD are women, who experience more pronounced cognitive decline than age‐matched men.^[^ ^3^, ^4^ ^]^

Microglia, the resident immune cells in the brain, play a crucial role in neuroinflammation and are strongly linked to the progression of neurodegeneration and synaptic dysfunction, especially in the context of AD. Their primary function is to remove cellular debris—such as damaged organelles, dead cells, and aggregated proteins—through phagocytosis and subsequent lysosomal degradation, which includes autophagy.^[^ ^5^, ^6^ ^]^ Autophagy is a cellular process that involves encapsulating intracellular debris within autophagosomes. The mammalian target of rapamycin complex 1 (mTORC1) pathway is a key regulatory pathway in autophagy, where its inhibition triggers the formation of autophagosomes.^[^ ^7^ ^]^ Microtubule‐associated protein 1 light chain 3 (LC3) is an essential factor in the formation and maturation of autophagosomal membranes.^[^ ^8^ ^]^ Once formed, these autophagosomes fuse with lysosomes to degrade their contents.^[^ ^9^ ^]^ The adaptor protein p62 plays a key role in this process by binding to ubiquitinated misfolded proteins and facilitating their incorporation into autophagosomes. During this process, p62 itself is also degraded, making its accumulation a widely recognized marker of impaired autophagic flux and defective clearance of autophagosomes.^[^ ^10^ ^]^

Recent studies have highlighted sex‐related differences in microglial characteristics. In a study using rat models, microglial colonization, morphology, and number were differentially affected by sex during development and adulthood.^[^ ^11^ ^]^ The expression of genes involved in proinflammatory responses also exhibits sex‐related differences, although the findings are controversial.^[^ ^12^, ^13^, ^14^ ^]^ Villa et al. demonstrated that female microglia exhibit a neuroprotective phenotype,^[^ ^12^ ^]^ whereas Hanamsagar et al. found that female microglia express more proinflammatory genes.^[^ ^14^ ^]^ Furthermore, transcriptomic analyses of microglia from male and female mice have revealed distinct sexual differentiation in adulthood, with more pronounced differences as age increases, potentially linking them to age‐related diseases such as AD.^[^ ^15^, ^16^ ^]^ Given the crucial role of microglial function in AD, these sex‐specific differences may play a significant role in the disparities in disease manifestation between men and women.

Sex‐based differences also influence autophagy induction, particularly in neurons, where females exhibit lower basal autophagy levels.^[^ ^17^ ^]^ However, the underlying mechanisms are not yet fully understood. Although some studies suggest that estrogen may activate autophagy, others report contradictory findings.^[^ ^17^ ^]^ Several autophagy‐related genes are located on the X chromosome; therefore, age‐related escape from X chromosome inactivation has been implicated in the reduction of basal autophagy observed in females. These sex‐related differences in autophagy may extend to microglia, potentially contributing to sex‐dependent disparities in susceptibility and progression of AD.^[^ ^17^ ^]^

The aging process differs between men and women, particularly in terms of gonadal hormones. The drastic decline in ovarian hormones during menopause contributes to female vulnerability to AD.^[^ ^18^, ^19^ ^]^ In contrast, men experience a more gradual decline in testosterone levels, which may play a protective role against AD.^[^ ^20^, ^21^ ^]^ However, the mechanisms by which testosterone influences microglial function and its implications for the sex‐based differences in AD pathology are not fully understood.

In this study, we investigated the role of testosterone in Aβ‐induced autophagy in microglia and its underlying mechanisms. Specifically, we explored testosterone signaling through its non‐genomic receptor G protein‐coupled receptor family C group 6 member A (GPRC6A), and its modulation of the mTORC1 signaling pathway to enhance autophagy, as a potential mechanism that may influence AD pathogenesis in males. We showed that testosterone‐mediated inhibition of GPRC6A signaling enhances autophagic flux in microglia, contributing to the clearance of Aβ. Our findings provide new information on the role of testosterone in modulating microglial autophagy, which may contribute to the lower susceptibility to AD observed in men.

## 2 Results

### 2.1 Sex‐Based Differences in Microglial Autophagy

**Figure 1:**
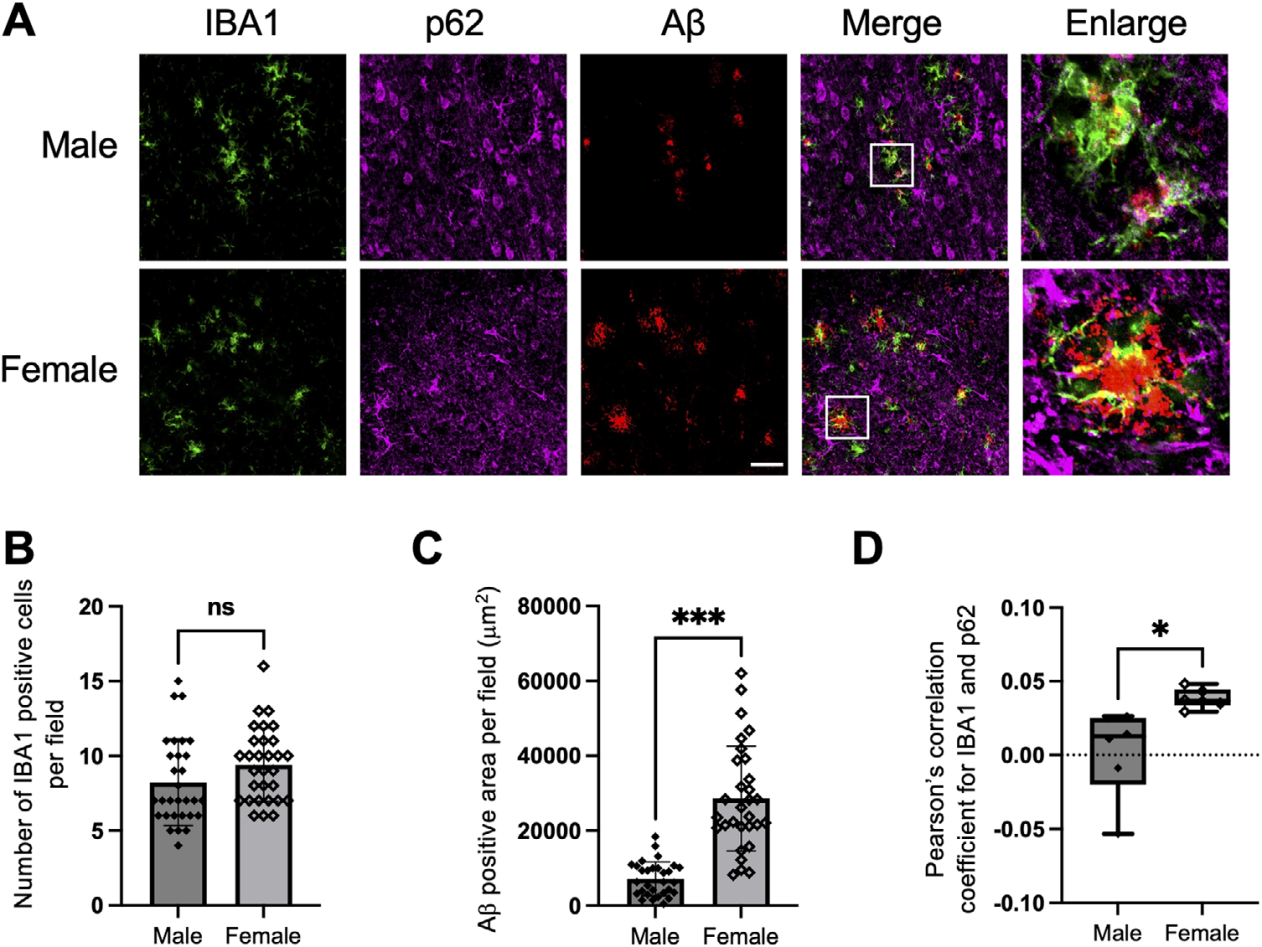
Sex‐based differences in microglial autophagy. A) Confocal images of IBA1 (green), p62 (a selective autophagy adaptor protein, magenta), and amyloid β (Aβ, red) in the cerebral cortex of 7‐month‐old 5xFAD mice. Scale bar: 50 µm. The panels on the far‐right show enlarged images of a white square area on the corresponding merged images. Thirty immunohistochemistry image sets were obtained from each of three male or three female mouse cerebral cortices. One representative image set is shown. Three additional sets are shown in Figure S1 (Supporting Information). B,C) Quantification of the number of microglia (B) and Aβ‐positive area (C). Each point represents the number (B) or area (C) per field. D) Pearson's coefficient indicating colocalization of p62 and IBA1. Data are presented as the mean ± SD (B–D, n = 30), ns: not significant. *p < 0.05, ***p < 0.001 by unpaired two‐tailed *t‐*test.

We first compared microglial autophagy between male and female 5xFAD mice, an animal model that rapidly develops severe amyloid pathology at 7 months of age when extracellular Aβ accumulation is pronounced.^[^ ^22^ ^]^ Immunostaining of the ionized calcium binding adaptor molecule 1 (IBA1), a microglia marker, in the cerebral cortex showed no significant differences in microglial numbers between the sexes (**Figure** **1**A,B; Figure S1, Supporting Information). Quantification of Aβ‐positive areas revealed that male mice exhibited significantly less Aβ accumulation in their brains compared to female mice (Figure 1A,C; Figure S1, Supporting Information). Given the lower association of microglia with Aβ plaques in males, we analyzed the colocalization of Aβ with p62 aggregates, indicative of autophagy deficiency,^[^ ^10^ ^]^ in IBA1‐positive microglial cells (far right images of Figure 1A). Pearson's correlation coefficient, indicating the colocalization of p62 and IBA1, revealed that male microglia had significantly lower levels of p62 than female microglia (Figure 1A,D). This colocalization suggests that Aβ undergoes autophagic degradation within microglia, with the process being elevated in the male microglia, potentially contributing to reduced plaque accumulation in the male brain.

### 2.2 Testosterone Enhanced Aβ‐Induced Autophagy in Microglial Cells

**Figure 2:**
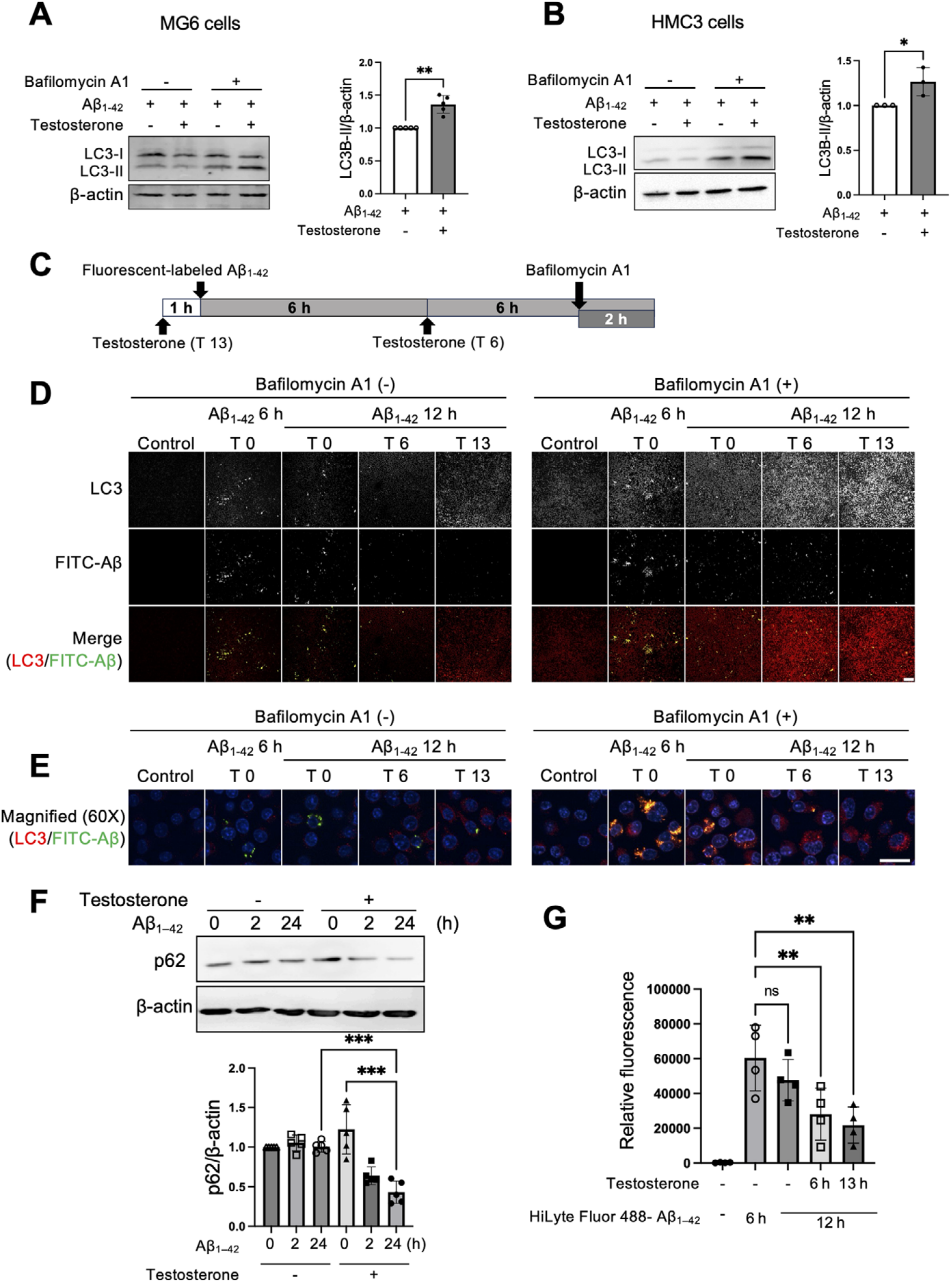
Testosterone enhances Aβ‐induced autophagy in microglial cells. A,B) MG6 cells (A) and HMC3 cells (B) were treated with Aβ_1–42_ fibrils for 24 h. The expression of LC3‐I and LC3‐II in whole‐cell lysates was assessed after 2 h of testosterone stimulation (100 nm). Bafilomycin A1 (100 nm) was added 30 min before testosterone treatment. Representative western blotting images of LC3 and loading control β‐actin (left) and the optical density quantification of LC3‐II relative to β‐actin in the presence of bafilomycin A1 (right graph of each) are shown. Experiments were repeated five (A) or three (B) times, and a representative set of images is shown in each case. Full membrane images for (A) and (B) are shown in Figure S2C,E (Supporting Information), and other sets of images are shown in Figure S2D,F (Supporting Information), respectively. C) Experimental design for MG6 cells exposed to fluorescent‐labeled Aβ_1‐42_ and testosterone as shown in (D) and (G). D,E) Representative immunofluorescence images of MG6 cells stimulated with FITC‐Aβ_1‐42_. The top row shows LC3‐positive puncta in grayscale, and the middle row shows Aβ‐positive areas, also in grayscale. The bottom row presents the merged images, with LC3 in red, FITC‐Aβ in green, and nuclei in blue (D). Magnified merged images are shown below the merged images (E). The experiments were repeated three times. Additional images are presented in Figure S2G (Supporting Information). Scale bars, 20 µm. F) MG6 cells were stimulated with Aβ_1–42_ fibrils for the indicated times in the presence or absence of testosterone. The expression of p62 was determined in whole‐cell lysates. The upper panel shows the representative western blot images of p62 and the loading control β‐actin, and the lower graph shows the optical density quantification of p62 relative to β‐actin. The experiments were repeated five times. Full membrane images and other images are shown in Figure S2H,I (Supporting Information). G) FACS analysis of MG6 cells treated without or with HiLyte Fluor 488‐labeled Aβ_1‐42_ for 6 or 12 h, with testosterone added either for the last 6 or 1 h before Aβ_1–42_ stimulation, and without testosterone. Analyses were repeated four times. Data are presented as the mean ± SD (A, n = 5; B, n = 3; D, n = 3, 6 images captured from 6 different fields of view per slide; F, n = 5; G, n = 4). ns: not significant. *p < 0.05, **p < 0.01, ***p < 0.001, for the indicated comparison, by unpaired two‐tailed *t‐*test (A, B) or by one‐way ANOVA followed by Tukey‐Kramer's HSD test (F, G).

Extracellular Aβ fibrils are known to induce autophagy in microglia.^[^ ^23^ ^]^ We confirmed that 24 h of Aβ_1–42_ stimulation significantly increased autophagic flux in the mouse microglial cell line MG6 (Figure S2A,B, Supporting Information). Autophagic flux was assessed by measuring the levels of phosphatidylethanolamine‐conjugated form of microtubule‐associated protein 1A/1B‐light chain 3 (LC3‐II) in the presence of bafilomycin A1, a V‐ATPase blocker that inhibits lysosome‐dependent degradation, leading to LC3‐II accumulation, a reliable marker of autophagic flux.^[^ ^24^ ^]^ To determine whether testosterone enhances Aβ‐induced autophagy, MG6 and HMC3 cells, a human microglial cell line, were treated with Aβ_1–42_ for 24 h, followed by the addition of testosterone or vehicle for the final 2 h. Testosterone treatment significantly increased LC3‐II levels in MG6 cells compared to vehicle‐treated cells (**Figure** **2**A; Figure S2C,D, Supporting Information). Similar results were observed in HMC3 cells (Figure 2B; Figure S2E,F, Supporting Information), suggesting that testosterone enhances autophagic activity in microglia.

To further investigate the role of testosterone in the autophagy process, we analyzed the number of LC3 positive puncta and their colocalization with fluorescein‐labeled Aβ_1–42_ in MG6 cells, following the experimental design shown in Figure 2C. In the absence of bafilomycin A1, FITC‐Aβ_1–42_ accumulation and LC3‐positive puncta were reduced due to degradation, compared to cells treated with bafilomycin A1 (Figure 2D, left panels). Low‐magnification images show that in the presence of bafilomycin A1, overall FITC‐Aβ_1–42_ accumulation and LC3‐positive cells were clearly observed at 6 h post‐treatment (Figure 2D, Aβ_1–42_ 6 h, T0). A high‐magnification image shows that intracellular FITC‐Aβ_1–42_ colocalize with LC3‐positive puncta (Figure 2E, Aβ_1–42_ 6 h, T0). While FITC‐Aβ_1–42_ accumulation gradually declined by 12 h (Figure 2D,E, Aβ_1–42_ 12 h, T0), LC3‐positive puncta remained elevated, indicating autophagy induction (Figure 2D,E; Figure S2G, Supporting Information). Testosterone treatment for 6 h increased the number of LC3‐positive puncta while reducing FITC‐Aβ_1–42_ accumulation in the presence of bafilomycin A1(Figure 2D,E, Aβ_1–42_ 12 h, T6). After 13 h of testosterone treatment, LC3‐positive puncta further increased, accompanied by a marked reduction in FITC‐Aβ_1–42_ accumulation (Figure 2D,E, Aβ_1–42_ 12 h, T13), supporting the notion that testosterone enhances Aβ‐induced autophagy.

Furthermore, we investigated p62 levels in MG6 cells after treatment with Aβ_1–42_ in the presence or absence of testosterone. Co‐stimulation with testosterone and Aβ_1–42_ resulted in a reduction of p62 levels at 2 h, with significant suppression at 24 h, whereas p62 levels remained unchanged when treated with Aβ_1–42_ alone (Figure 2F; Figure S2H,I, Supporting Information). These results indicate that testosterone enhanced Aβ‐induced autophagy in microglia.

To confirm whether testosterone promotes Aβ clearance, we conducted a fluorescence‐activated cell sorting (FACS) analysis. MG6 cells were treated with HiLyte Fluor 488‐labeled‐Aβ_1–42_ to track internalized Aβ. The uptake of Aβ_1–42_ in MG6 cells peaked at 6 h after treatment, as indicated by the relative fluorescence of HiLyte Fluor 488‐labeled Aβ_1–42_ in the cells. Although not statistically significant, a tendency to decrease was observed 12 h after treatment. Testosterone accelerated this Aβ clearance process when administered during the final 6 h of the 12‐h Aβ_1–42_ treatment (Figure 2G). An even greater reduction in Aβ_1–42_ levels was observed when testosterone was added 1 h before HiLyte Fluor 488‐labeled Aβ_1–42_ stimulation and was maintained throughout the 12 h treatment. These results suggest that testosterone‐mediated autophagy promotes Aβ clearance.

### 2.3 Testosterone Promoted Autophagy by Inhibiting the GPRC6A‐Mediated ERK‐mTOR Signaling Pathway

**Figure 3:**
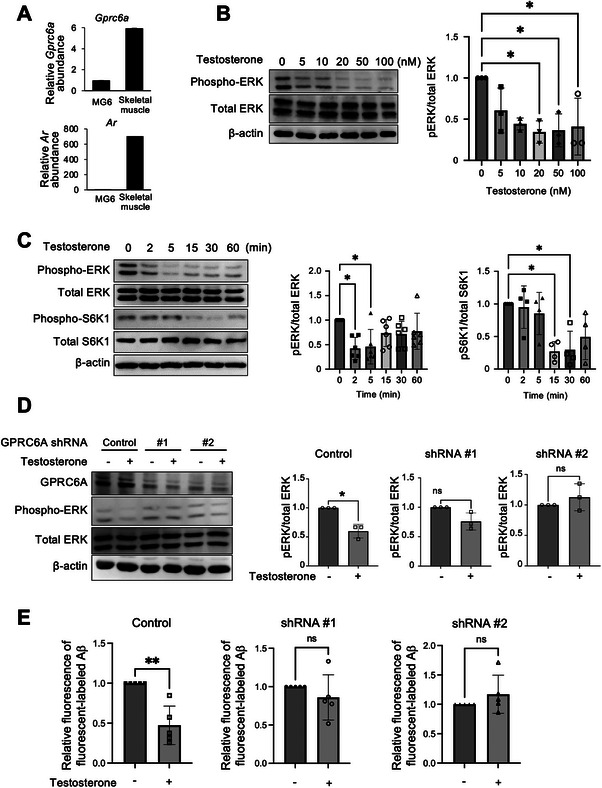
Testosterone promotes autophagy by inhibiting the GPRC6A‐mediated ERK/mTOR signaling pathway. A) Relative mRNA expression of *Ar* (upper) and *Gprc6a* (lower). Skeletal muscle was used as positive control. B) Western blot analysis of total and phosphorylated forms of ERK in MG6 cells stimulated with testosterone for 1 h at different concentrations. Representative blots (left images) and the relative phosphorylated/total protein ratio for ERK (right graph) are shown. C) Western blot analysis of total and phosphorylated forms of ERK and S6K1 in MG6 cells stimulated with 20 nm of testosterone for the indicated times. Representative blots (left images) and the relative phosphorylated/total protein ratio for ERK and S6K1 (right graphs) are shown. D) GPRC6A was silenced in MG6 cells using shRNAs #1 and #2 and treated with 100 nm testosterone for 2 h. Representative blots and the relative phosphorylated/total for ERK are shown. E) FACS analysis of control and GPRC6A‐silenced MG6 cells treated with HiLyte Fluor 488‐labeled Aβ_1‐42_ for 12 h, with or without testosterone added 1 h before Aβ_1–42_ stimulation. Analyses were repeated five times. Data are presented as the mean ± SD (A and D, n = 3; B, C, and E, n = 5). ns: not significant. *p < 0.05, for indicated comparisons, by one‐way ANOVA followed by Tukey‐Kramer's HSD test (B,C) or by unpaired two‐tailed *t‐*test (D,E).

Next, we investigated the signaling pathway by which testosterone enhances Aβ‐induced autophagy in microglia. Testosterone can signal through two types of receptors: the classical nuclear androgen receptor (AR), which mediates genomic effects as a transcription factor, and the non‐genomic membrane receptor GPRC6A, which is activated by a variety of ligands including amino acids in addition to testosterone, and is implicated in the regulation of autophagy.^[^ ^25^, ^26^ ^]^ To determine which pathway is involved, we first examined the expression of AR and GPRC6A. The gene expression of *Ar* in MG6 cells was very low, confirmed by comparison with its expression levels in skeletal muscle, whereas *Gprc6a* was substantially expressed in MG6 cells (**Figure** **3**A).

GPRC6A activation regulates the AKT and extracellular signal‐regulated kinase (ERK) 1/2 signaling pathways,^[^ ^25^, ^27^, ^28^ ^]^ both of which serve as upstream regulators of the mTORC1 pathway by inhibiting the tuberous sclerosis complex (TSC).^[^ ^29^ ^]^ Notably, suppression of mTORC1 pathway promotes autophagy.^[^ ^30^ ^]^ To investigate the involvement of this pathway, we treated MG6 cells with testosterone and examined ERK and AKT signaling. Testosterone dose‐dependently suppressed ERK‐1/2 phosphorylation without affecting total ERK levels, with 20 nm testosterone being sufficient to inhibit ERK phosphorylation (Figure 3B; Figure S3A, Supporting Information). This inhibition occurred rapidly within 2 min of testosterone treatment (Figure 3C; Figure S3B, Supporting Information). However, testosterone did not affect AKT phosphorylation at either T308 or S473 (Figure S3C,D, Supporting Information). Additionally, testosterone suppressed the phosphorylation of p70 S6 kinase (S6K1), a downstream target of mTORC1, indicating that testosterone suppresses mTORC1 activity (Figure 3C; Figure S3E, Supporting Information).

To further confirm the involvement of GPRC6A signaling, we transiently silenced GPRC6A using two independent shRNAs. Testosterone failed to suppress the phosphorylation of ERK1/2 when GPRC6A expression was transiently silenced (Figure 3D; Figure S3F,G, Supporting Information). Furthermore, clearance of internalized HiLyte Fluor 488‐labeled‐Aβ_1–42_, assessed by FACS analysis, revealed that the addition of testosterone accelerated Aβ_1–42_ clearance in cells transfected with control shRNA but had no effect in GPRC6A‐knockdown cells (Figure 3E). These results demonstrate that the effect of testosterone on suppressing the ERK signaling pathway and subsequent clearance of Aβ_1–42_ is mediated through GPRC6A.

### 2.4 Testosterone Deprivation by Testicular Removal Increased Aβ Accumulation in the Brain of 5xFAD Mice

**Figure 4:**
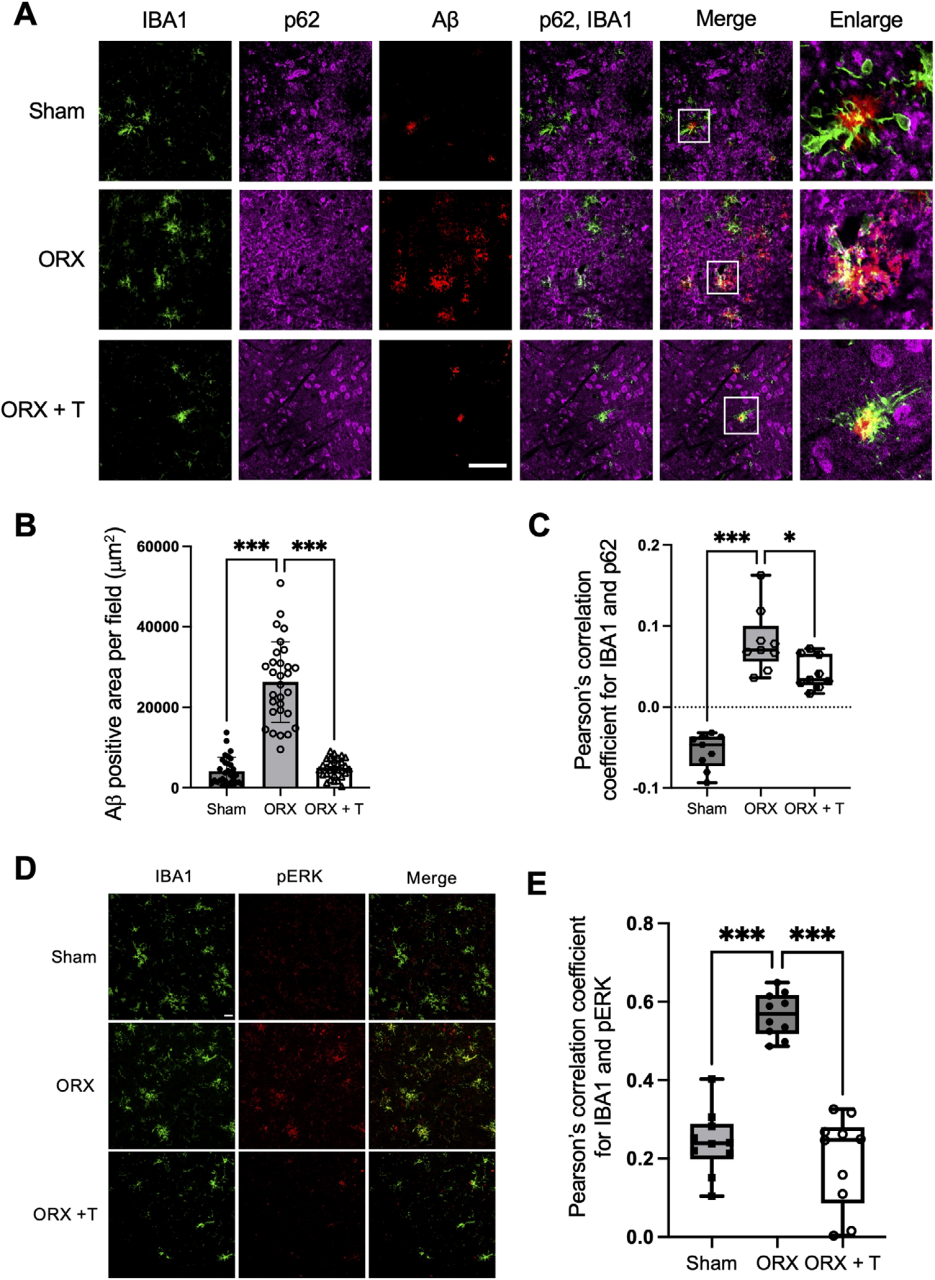
Testosterone deprivation by testicular removal increases Aβ in the brain of 6‐month‐old 5xFAD mice. A) Confocal images of IBA1 (green), p62 (magenta), and Aβ (red) in the cortex of 6‐month‐old 5xFAD mice that underwent sham surgery (Sham), orchidectomy (ORX), or orchidectomy with testosterone supplementation (ORX + T). Thirty immunohistochemistry image sets were obtained from cerebral cortices of each group. One representative image set is shown. Three additional sets are shown in Figure S4A (Supporting Information). Scale bar: 100 µm. B) Quantification of Aβ‐positive area per field from the image shown in (A). Each point represents the area per field. C) Pearson's correlation coefficient indicating colocalization of IBA1 and p62. D) Confocal images of IBA1 (green) and phosphorylated ERK (red) in the cortex of Sham, ORX, and ORX + T groups. One representative image set is shown. Three additional sets are shown in Figure S4B (Supporting Information). Scale bar: 100 µm. E) Colocalization of IBA1 and phosphorylated ERK in (D) were quantified. Data are presented as the mean ± SD. *p < 0.05, ***p < 0.001 for indicated comparisons, by one‐way ANOVA followed by Tukey–Kramer's HSD test (B,C) or by unpaired two‐tailed *t‐*test (E).

To investigate the role of testosterone in regulating autophagic activity in microglia, we performed orchidectomy (ORX) on 4‐month‐old 5xFAD male mice and compared Aβ accumulation and microglial autophagy. We used 4‐month‐old mice for this experiment, as extracellular Aβ accumulation begins around 2 months of age in 5xFAD mice and is expected to remain at a relatively low level at 4 months.^[^ ^22^ ^]^ Using this age, we aimed to evaluate the effects of testosterone during the early phase of amyloid pathology, when its impact on Aβ burden can be observed more effectively. Five weeks after ORX, we observed a marked increase in Aβ plaque accumulation compared with sham‐operated mice (**Figure** **4**A,B; Figure S4A, Supporting Information). Furthermore, the number of microglia expressing p62 was significantly higher in the ORX group (Figure 4A,C). Importantly, testosterone supplementation in ORX mice reversed Aβ accumulation and was accompanied by a significant reduction in p62‐positive microglia compared to the ORX group (Figure 4A–C).

The relevance of testosterone‐mediated ERK signaling was also analyzed by assessing ERK phosphorylation levels in ORX and sham‐operated wild‐type C57BL/6J mice. Brain slices from both groups were stained for phosphorylated ERK and IBA1, revealing a significant increase in ERK phosphorylation in cells positive for IBA1 in ORX mice (Figure 4D, upper and middle panels; Figure S4B, Supporting Information). Furthermore, the upregulated ERK phosphorylation was restored to the levels observed in the sham‐operated group upon testosterone supplementation (Figure 4D, bottom panels; Figure S4B, Supporting Information). These results suggest that circulating testosterone suppresses the ERK signaling pathway in microglia. Collectively, these findings suggest that testosterone regulates autophagy by inhibiting the GPRC6A/ERK signaling pathway, thereby enhancing microglial autophagic activity and Aβ clearance.

### 2.5 Histological Evaluation of Microglial Autophagy in Plaque‐Associated Microglial Cells in the Cortex of Male and Female Patients with AD

**Figure 5:**
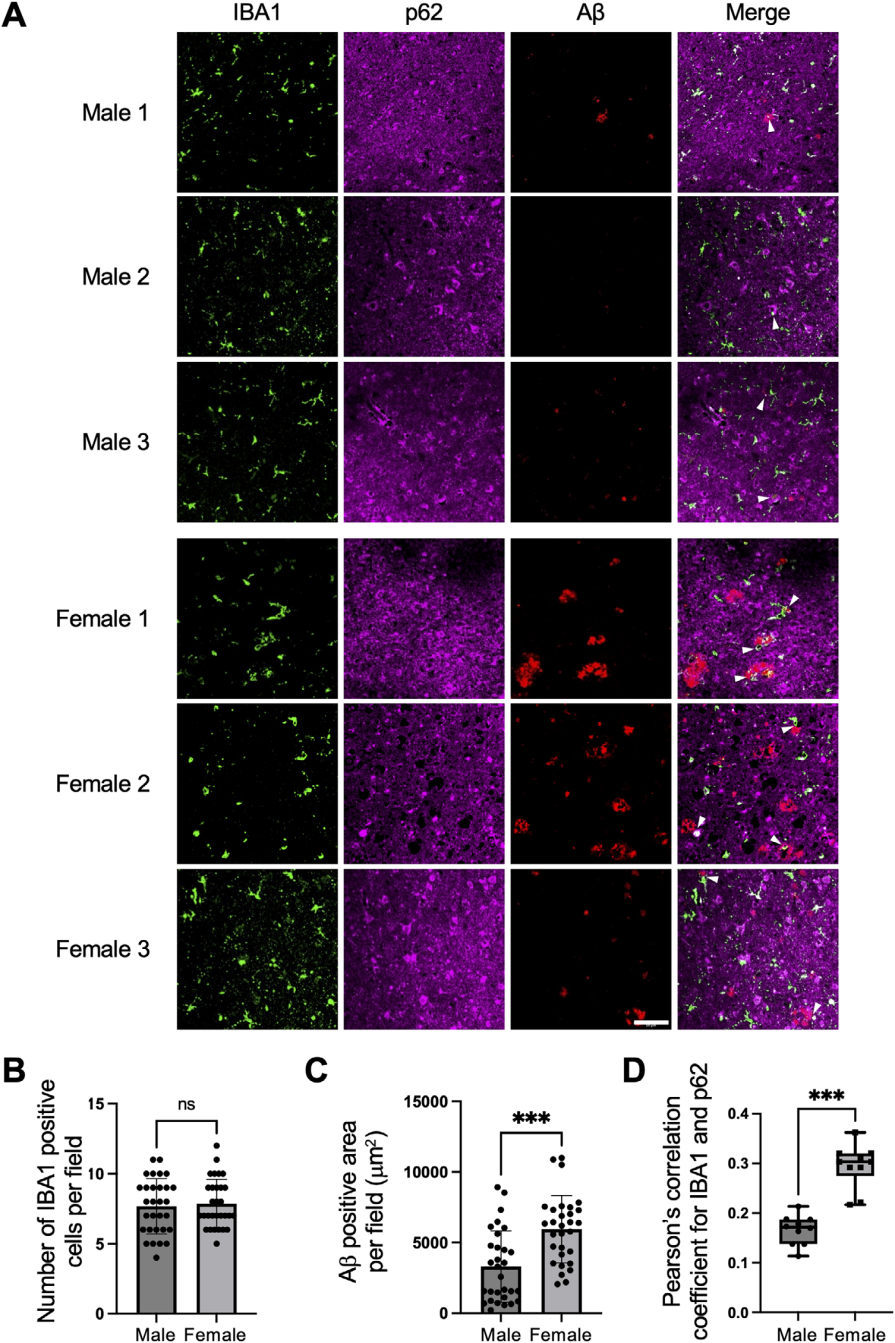
Histological evaluation of microglial autophagy in plaque‐associated microglial cells in the cortex of male and female patients with Alzheimer's disease (AD). A) Confocal images of IBA1 (green), p62 (magenta), and Aβ (red) in the cortex of male and female patients with AD. One representative image set is shown. Three additional sets are shown in Figure S5 (Supporting Information). Scale bar: 100 µm. B,C) Quantification of the number of microglia (B) and Aβ‐positive area (C) from the images in (A). D) Pearson's coefficient, indicating colocalization of p62 and IBA1. Data are presented as the mean ± SD (n = 3; 20 images captured from 20 different fields of view per slide). ns: not significant. ***p < 0.001 for indicated comparisons, by one‐way ANOVA followed by Tukey–Kramer's HSD test.

Finally, we analyzed postmortem brain tissue slices from male and female patients with AD to determine whether human microglia exhibit similar associations with Aβ and autophagic activity as observed in mouse AD models. Immunofluorescence staining was performed using anti‐p62, anti‐IBA1, and anti‐Aβ antibodies (**Figure** **5**A; Figure S5, Supporting Information). Although the number of IBA1‐positive microglial cells did not differ between males and females (Figure 5B), Aβ accumulation was significantly higher in female brain sections (Figure 5C). Some IBA1‐positive microglial cells were closely associated with Aβ plaques (arrowheads in Figure 5A merge images), indicating their involvement in Aβ clearance. The colocalization of Aβ with p62 aggregates in IBA1 positive microglial cells was more pronounced in women, mirroring the observation in 5xFAD mice. Pearson's correlation coefficient analysis of p62 and IBA1 colocalization revealed higher levels of p62 in female microglia, indicating suppressed autophagic activity (Figure 5D). These observations indicate that higher levels of testosterone in the male brain may improve autophagy, thereby aiding microglia to clear Aβ more effectively.

## 3 Discussion

**Figure 6:**
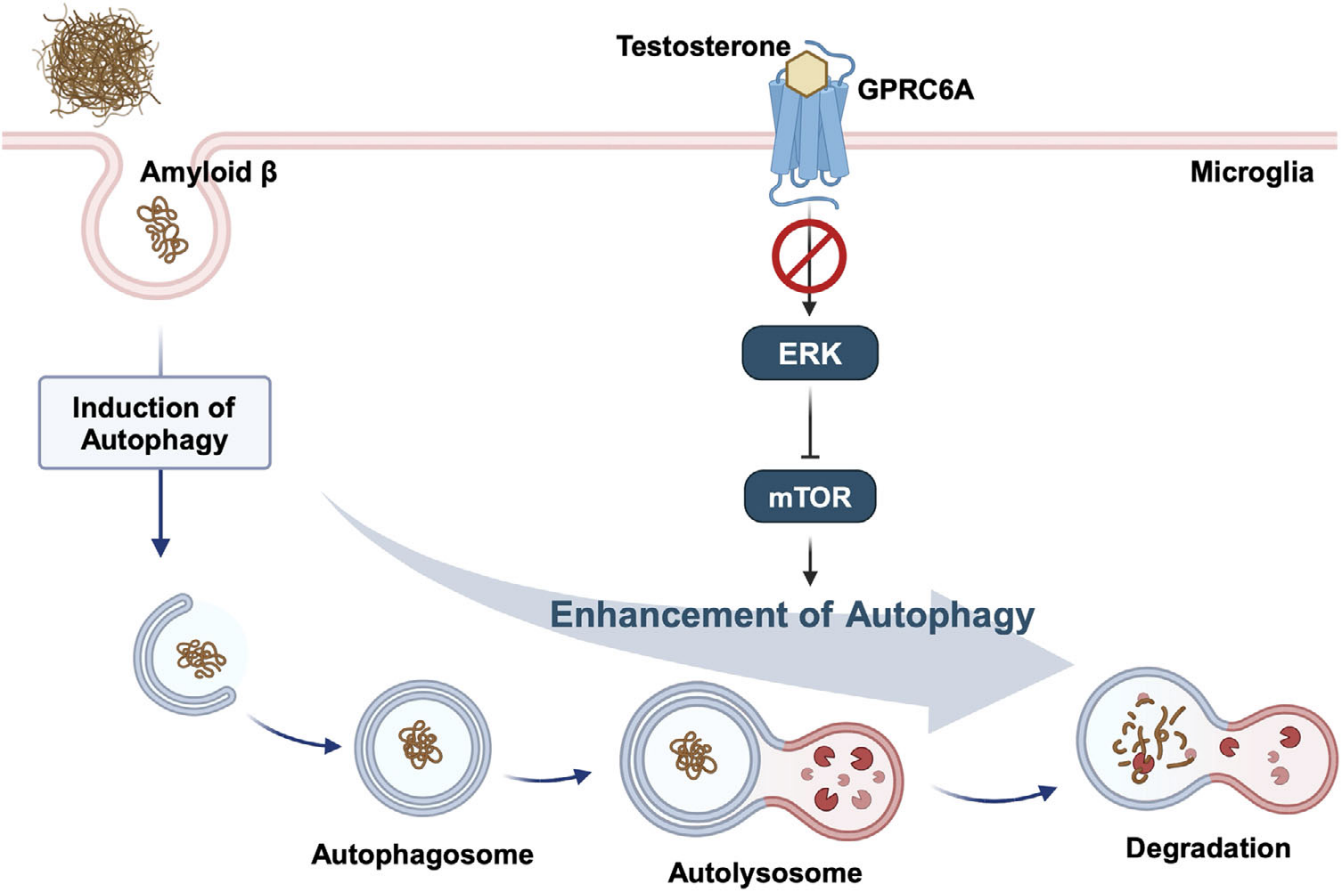
Hypothetical schematic representation of the testosterone‐GPRC6A signaling pathway that enhances autophagic activity in male microglia. Extracellular Aβ induces autophagy in microglia. Testosterone inhibits the ERK signaling pathway via GPRC6A, which in turn suppresses the mTOR activity and promotes Aβ‐induced autophagy. This testosterone‐mediated inhibition of the GPRC6A/ERK/mTOR axis may contribute to lower susceptibility to AD in men. This figure was created with BioRender.com.

In this study, we demonstrated that testosterone improves microglial autophagy by suppressing the ERK/mTOR signaling pathway via GPRC6A, leading to the degradation of extracellular Aβ (**Figure** **6**). In particular, brain sections of 5xFAD mice showed that autophagy is suppressed in female microglia, correlating with higher Aβ accumulation. This suppression was similarly observed in brain sections of patients with AD, emphasizing a comparable reduction in autophagy in human female microglia. These findings indicate that testosterone‐mediated inhibition of the ERK signaling pathway enhances Aβ‐induced autophagy in microglia, possibly contributing to the lower susceptibility to AD observed in men compared with women.

Although some studies support the neuroprotective effects of estrogen,^[^ ^31^, ^32^ ^]^ hormone replacement therapy in patients with AD has not shown positive effects on cognitive function in postmenopausal women,^[^ ^33^, ^34^, ^35^ ^]^ despite being effective in certain populations with at‐risk *APOE4* carriers.^[^ ^36^ ^]^ Conversely, testosterone reportedly has neuroprotective effects.^[^ ^20^, ^37^, ^38^ ^]^ A clinical study investigating the effect of dehydroepiandrosterone, which significantly increases plasma testosterone levels, showed cognitive improvement in postmenopausal women with mild to moderate cognitive impairment.^[^ ^39^ ^]^ In our study, consistent with other studies,^[^ ^40^ ^]^ we observed that Aβ accumulation was lower in male patients with AD compared to females, likely due to higher circulating testosterone levels in males. Consistently, our findings also showed that female microglia had higher levels of p62, suggesting suppressed autophagy, which may contribute to the increased Aβ pathology observed in females. Although estrogen deficiency probably contributes to sex differences in AD, our findings suggest that sustained testosterone levels maintained in males throughout life may play a more critical role in modulating the onset of AD. To investigate this hypothesis, we performed ORX surgery on 4‐month‐old 5xFAD mice, a stage when Aβ accumulation is relatively low. We observed a significant increase in Aβ accumulation in ORX mice 5 weeks post‐surgery. These findings support the notion that sustained testosterone levels may contribute to the lower susceptibility to AD observed in men.

Accumulating evidence indicates that the autophagy‐lysosomal pathway is a key mechanism for clearing aggregated proteins such as Aβ plaques. A study using mouse models lacking microglial autophagy has demonstrated that microglial autophagy is essential to engaging and degrading Aβ plaques, thereby mitigating neuroinflammation.^[^ ^41^ ^]^ Furthermore, the autophagy‐lysosomal pathway in microglia exerts a protective role against AD by degrading aggregated extracellular proteins.^[^ ^23^, ^42^, ^43^ ^]^ For example, Cho et al. reported that extracellular Aβ fibrils can induce autophagy in microglia, and impairment of this process leads to activation of the NOD‐like receptor family pyrin domain containing 3 (NLRP3) inflammasome, compromising neuronal survival.^[^ ^23^ ^]^ Additionally, a deficiency in microglial autophagy has also been linked to increased spreading of tau and exacerbated tau pathology in tauopathy mouse models.^[^ ^43^ ^]^ Hyperphosphorylated tau, primarily produced in neurons, can spread from degenerating neurons, where it may be internalized by microglia for degradation or transferred to other neurons, further driving neurodegeneration.^[^ ^44^ ^]^ Therefore, lower circulating testosterone levels in females may contribute to reduced autophagic activity in microglia compared with males, potentially impairing their ability to clear aggregated proteins such as Aβ.

Androgen reportedly suppresses autophagy in Sertoli cells and the human prostate cancer cell line LNCap through its nuclear AR.^[^ ^45^, ^46^ ^]^ In microglia, AR expression levels are significantly low,^[^ ^47^ ^]^ despite the expression of the non‐genomic testosterone receptor GPRC6A, suggesting that testosterone‐GPRC6A signaling may be the dominant signaling pathway. GPRC6A activates ERK signaling upon stimulation by various ligands, including L‐α‐amino acids such as arginine and ornithine, bone‐derived osteocalcin, and testosterone.^[^ ^26^, ^28^, ^48^, ^49^ ^]^ Arginine activates GPRC6A and its downstream ERK and AKT pathways, leading to autophagy suppression in neuronal cells, exacerbating tau pathology.^[^ ^25^ ^]^ In this context, GPRC6A senses nutrient‐sufficient states to regulate autophagic activity. Our study demonstrated that testosterone stimulation suppressed basal ERK phosphorylation, potentially mediated by amino acids, thereby facilitating activation of microglial autophagy under basal conditions. This was further supported by findings that testosterone reduction by ORX surgery led to increased basal ERK phosphorylation in brain sections of male WT mice, which was restored to the levels of sham‐operated mice by testosterone supplementation. As previously reported, testosterone stimulation of GPRC6A can activate ERK and mTOR signaling in prostate cancer cells and HEK‐293 cells overexpressing GPRC6A.^[^ ^50^, ^51^ ^]^ Given the discrepancies in phenotypes observed across various GPRC6A knockout mouse strains, it is likely that the response of GPRC6A to its ligands varies depending on the cell type.^[^ ^26^, ^52^ ^]^ Further research is needed to clarify how GPRC6A signaling impacts different cell types. Although GPRC6A can activate the ERK signaling pathway in some cells, our findings indicate that in microglia, testosterone suppresses the GPRC6A‐ERK pathway, likely due to cell type‐specific factors.

There are limitations in our study. One limitation is that we were unable to use specific genetic models, such as GPRC6A‐deficient or conditional knockout mice, to further validate that testosterone promotes Aβ reduction through inhibition of the GPRC6A‐mediated ERK‐mTOR pathway. Since the GPRC6A‐ERK signaling pathway is activated by multiple ligands, selecting appropriate mouse models and designing precise experiments will be crucial for future studies. Second, we could not obtain data on circulating testosterone levels in patients with AD, which prevented us from directly correlating our findings with human testosterone status and Aβ pathology. Future studies incorporating data on patients’ testosterone levels will be important to strengthen the clinical relevance of our findings.

## 4 Conclusion

Our study demonstrates the crucial role of testosterone in enhancing autophagic activity in microglia by inhibiting GPRC6A‐mediated ERK signaling, thereby facilitating the clearance of Aβ plaques. Higher testosterone levels in males can contribute to enhanced autophagy and reduced Aβ accumulation, potentially explaining the lower susceptibility to AD observed in males. These findings suggest that inhibiting the GPRC6A pathway, similar to the effects of testosterone, could offer therapeutic strategies for AD.

## 5 Experimental Section

### Reagents

Amyloid β‐protein_1–42_ (Aβ_1–42_) (Peptide Institute Inc., Osaka, Japan) was dissolved in dimethyl sulfoxide (DMSO) to a final concentration of 0.5 mm and stored at ‐20 °C until use. Aβ_1–42_ was diluted to 50 µm with Hanks’ balanced salt solution (HBSS) buffer and incubated for 24 h at 37 °C to prepare Aβ enriched in fibrils. Aβ_1–42_, HiLyte Fluor 488‐labeled (AS‐60479‐01, AnaSpec, Drive Fremont, CA, USA) was dissolved in 1% NH_4_OH to a final concentration of 400 µm and stored at ‐80 °C until use and was diluted to 100 µm with PBS and incubated at 37 °C for 1 h prior to use. Bafilomycin A1 (Selleck Chemicals, Houston, TX, USA) was diluted in DMSO. Testosterone (A7703, Shifeng biol, Shanghai, China; and T1500, Sigma‐Aldrich, St. Louis, MO, USA) was dissolved in 100% ethanol.

### Animals

AD mouse model 5xFAD transgenic mice and C57BL/6J mice were provided by Jackson Laboratory (Bar Harbor, Maine, USA). All animal procedures were approved by the institutional animal care and use committees of the Beijing Institute of Technology (approval no. BIT‐EC‐SCXK2022‐0016‐M‐041) and Kyushu University (approval no. A21‐136) and were conducted in accordance with all relevant ethical regulations for animal testing and research. All mice were housed under light/dark conditions at 23 ± 1 °C for 12‐h cycles. The mice were fed standard rodent food (Keao Xieli Feed Co. Ltd., Beijing, China) and water. For the analysis of microglial autophagy in 5xFAD mice, 3 male and 3 female 7‐month‐old 5xFAD mice were each used for immunostaining experiments. For ORX experiments, 12 mice at 4 months of age were randomly divided into two groups and underwent orchidectomy (ORX, 8 mice) or sham surgery (4 mice). The ORX group was divided into two subgroups for the testosterone supplementation experiment. Half of the mice were injected subcutaneously with testosterone (3 µg/g body) daily (at 9 a.m.) for 4 weeks, beginning 5 days after recovery from ORX surgery. The other half was injected with vehicle (10% ethanol, 90% sesame oil). All surgeries were performed under anesthesia with a combination of medetomidine (0.3 mg kg^−1^), midazolam (4.0 mg kg^−1^), and butorphanol (5.0 mg kg^−1^). At the end of the treatment period, mice were transcardially perfused with saline (0.9% NaCl) followed by 4% paraformaldehyde.

### Brain Section Preparation and Immunohistochemistry

The brains extracted from the perfused mice were immediately immersed in a 10‐mL tube containing 4% paraformaldehyde after fixation at 4 °C for 24 h. The post‐fixed brain tissue was then subjected to gradient dehydration using 30% sucrose prior to embedding and freezing in Tissue‐Tek optimal cutting temperature compound (Sakura Finetek Japan, Tokyo, Japan). Sectioning was performed at a thickness of 20 µm using Minux FS800A cryostats (RWD Life Science, Shenzhen, China). Slices were stored at ‐20 °C until use. Before staining, sections were washed three times with PBS and then blocked with PBS containing 0.3% Triton X‐100 (PBST) with 10% donkey serum for 2 h at 20–25 °C. After blocking, sections were incubated with the primary antibody in PBST with 10% donkey serum at 4 °C overnight. Following three 5‐min washes in PBS, sections were incubated with secondary antibody in PBST for 2 h at 20–25 °C. Nuclei were stained with Hoechst stain. Images were acquired using an LSM980 confocal microscope (Carl Zeiss, Oberkochen, Germany) and processed using Fiji software 2.14.0 (National Institute of Health, Bethesda, MD, USA).

### Cell Culture

MG6 cells, a mouse microglial cell line, were obtained from Riken Cell Bank (RCB2403, Ibaraki, Japan), and HMC3 cells, a human microglial cell line, were obtained from the American Type Culture Collection (CRL‐3304, Manassas, VA, USA). Cells were cultured in Dulbecco's modified Eagle Medium (D‐MEM with high glucose; Fujifilm Wako Pure Chemical, Tokyo, Japan) containing 10% fetal bovine serum (FBS, 10270106, Gibco, Waltham, MA USA), insulin (10 µg mL^−1^, Cell Science & Technology Institute Inc., Sendai, Japan), and 0.1 mm 2‐mercaptoethanol (Sigma‐Aldrich), in a 37 °C incubator, 5% CO_2_. Testosterone dissolved in 100% ethanol was added to the culture medium containing charcoal‐stripped FBS (Serana, Brandenburg, Germany) at a final ethanol concentration of 0.1%.

### Immunocytochemistry

Cells were seeded on glass coverslips in a 12‐well plate and stimulated with 0.5 µm FITC‐Aβ_1‐42_ for 12 h. Testosterone was added 1 h before FITC‐Aβ_1‐42_ treatment, or 6 h before fixation. Bafilomycin A1 at 100 nm was added for the final 2 h. The cells were then fixed in 100% methanol for 15 min at ‐20 °C. Subsequently, cells were blocked with 0.3% Triton X‐100/PBS/1% BSA for 1 h. After blocking, cells were incubated with the primary antibody in PBST at 4 °C overnight. After three 5‐min washes in PBS, cells were incubated with the secondary antibody in PBST for 1 h at room temperature. Nuclear staining was performed using DAPI (Southern Biotech, Birmingham, AL, USA). Images were acquired using Nikon AX‐NiE/NS confocal microscope (Nikon, Tokyo, Japan). The antibodies used are listed in Table S1 (Supporting Information).

### Western Blotting

Cultured cells were washed three times with ice cold PBS and lysed in RIPA buffer (Fujifilm Wako Pure Chemical) supplemented with protease and phosphatase inhibitor cocktail (Nacalai Tesque Inc., Kyoto, Japan). Cell lysates were centrifuged at 21,500 × *g* for 10 min at 4 °C. Total lysates (10 µg protein) were run on sodium dodecyl sulfate‐polyacrylamide gel electrophoresis gels and transferred to polyvinylidene difluoride membranes. After blocking with Blocking One (Nacalai Tesque) for total protein and Blocking One‐P (Nacalai Tesque) for phosphorylated protein, membranes were incubated with a primary antibody at 4 °C overnight, followed by testing with secondary antibodies for 1 h at 20–25 °C. The antibodies used are listed in Table S1 (Supporting Information). Proteins were detected using ImmunoStar LD (Fujifilm Wako) and visualized with ImageQuant LAS 4000 mini (GE Healthcare, Chicago, IL, USA). Band intensity was determined using ImageJ 1.53a analysis software (free software National Institutes of Health).

### Flow Cytometry Analysis

For Aβ_1–42_ uptake determinations, MG6 cells were seeded in 6‐well plates and incubated with 0.5 µm HiLyte Fluor 488‐labeled Aβ_1‐42_ for 12 h. Testosterone was added 1 h before Aβ_1–42_ treatment, or 6 h before the end of the experiment. Cells were washed once with 2 mL of PBS, then removed by incubation with 0.03% Trypsin (without EDTA) for 1 min at 37 ° C. The cells were then mixed with 2 mL of PBS, centrifuged at 380 × *g* for 5 min, and the supernatant was removed. The cell pellet was resuspended in 0.5 mL of PBS, centrifuged again at 380 × *g* for 5 min, and transferred to a 1.5 mL microcentrifuge tube. Finally, the supernatant was transferred to a new flow cytometer tube and tested within 1 h at 20–25 °C. The samples were analyzed using a BD FACSAria III cytometer (BD Biosciences, San Jose, CA, USA) or BD FACSLyric (BD Biosciences). MG6 cells were gated based on side‐scattering (SSC) versus forward‐scattering (FSC) height plots, and the fluorescence intensity of HiLyte Fluor 488‐labeled Aβ_1‐42_ within the gate was detected at the excitation wavelength of 488 nm by flow cytometry. The FACS data were analyzed using FlowJo software 10.8.1 (Tree Star, Inc., Ashland, OR, USA).

### RNA Extraction and qPCR

Total RNA was extracted from MG6 cells or mouse skeletal muscle using a ReliaPrep RNA Tissue Miniprep kit (Promega, Madison, WI, USA) according to the manufacturer's protocol, and reverse transcription was performed using the High‐Capacity cDNA reverse transcription kit (Thermo Fisher Inc., Waltham, MA, USA). The resulting cDNA was subjected to quantitative PCR analysis with THUNDERBIRD Next SYBR qPCR Mix (Toyobo, Osaka, Japan) and a StepOnePlus instrument (Applied Biosystems, Foster City, CA, USA). Primer sequences were obtained from PrimerBank (https://pga.mgh.harvard.edu/primerbank/index.html):

*Ar*, 5′‐TCCAAGACCTATCGAGGAGCG‐3′ (forward) and 5′‐ GTGGGCTTGAGGAGAACCAT‐3′, PrimerBank ID 118129906c1.

*Gprc6a*, 5′‐ ATCCATCGCGGTCTCAAGGA‐3′ (forward) and 5′‐ AAGGAAAGCGGATCTTGTCAC‐3′ (reverse), PrimerBank ID 23346474c3.

*Actb*, 5′‐GGCTGTATTCCCCTCCATCG‐3′ (forward) and 5′‐ CCAGTTGGTAACAATGCCATGT‐3′ (reverse), PrimerBank ID 6671509a1.

### Short Hairpin RNA Plasmids

The bacteriologic glycerol stock of validated short hairpin RNA (shRNAs) for Gprc6a knockdown, along with an shRNA insert and a control without an shRNA insert, were purchased from Merck (MISSION shRNA, RefSeq: NM_153071; Rahway, NJ, USA). The target sequences were: #1 (TRCN0000026076) 5′‐CCCTGTCTATACTACCACATT‐3′; #2 (TRCN0000026134): 5′‐CCGGGACTCATTTATAGCATT‐3′. ShRNA plasmids were transfected using Lipofectamine 3000 (Thermo Fisher Scientific).

### Human Brain Samples

Post‐mortem frontal cortex tissues from three sets of male and female patients with AD were selected from the National Health and Disease Human Brain Tissue Resource Center. The patients’ sexes and ages were as follows: male, 82 years old; male, 87 years old; male, 73 years old; female, 86 years old; female, 84 years old; and female, 82 years old. All patients signed informed consent forms and agreed to use their brain materials for medical research. In this study, the principle of using human brain samples in the Declaration of Helsinki was strictly observed. The use of the study samples was approved by the Institutional Animal Care and Use Committees of Beijing Institute of Technology (human brain sample using license: BIT‐EC‐H‐20211191).

### Statistical Analyses

All data analysis and statistics were calculated using the GraphPad Prism v.9.5.0 package (GraphPad Software, San Diego, CA, USA). Two groups were compared using an unpaired *t*‐test. In more than two experimental groups, data were analyzed by two‐way ANOVA with a post hoc Tukey's test. *P* values < 0.05 were considered statistically significant.

## Conflict of Interest

The authors declare no conflict of interest.

## Author Contributions

A.M. conceived and designed the experiments. H.D. performed most of the experiments and analyzed the data. J.N. and T.K. helped design the experiments. H.D. and S.Z. performed animal experiments. H.D., A.M., and T.K. wrote the manuscript. J.N. and I.T. provided suggestions for experiments and data analysis. Y.Y., T.S., E.J., I.T., and T.K. provided technical support, contributed to the discussion, and reviewed the article. All authors read and approved the final manuscript.

## Supporting information



Supporting Information

## Data Availability

The data that support the findings of this study are available in the supplementary material of this article.
