# Supplementary material for: Role of Testosterone Signaling in Microglia: A Potential Role for Sex‐Related Differences in Alzheimer's Disease
Source: Adv Sci (Weinh). 2025 Mar 24;12(19):2413375. doi: 10.1002/advs.202413375 (PMC12097063; doi:10.1002/advs.202413375)
Supplement: Supplementary file 1 — Supporting Information [file ADVS-12-2413375-s001.docx]

Supporting Information

Role of testosterone signaling in microglia: a potential role for sex differences in Alzheimer's disease

Haiyan Du^1^, Akiko Mizokami^2^*, Junjun Ni^3^, Simeng Zhang^3^, Yosuke Yamawaki^4^, Tomomi Sano^1^, Eijiro Jimi^2,5^, Isei Tanida^6^, Takashi Kanematsu^1^*

*** Corresponding authors**

**Akiko Mizokami**

OBT Research Center, Faculty of Dental Science, Kyushu University, 3-1-1 Maidashi, Higashi-ku, Fukuoka 812-8582, Japan, Email: akiko-k@dent.kyushu-u.ac.jp

**Takashi Kanematsu**

Department of Cell Biology, Aging Science, and Pharmacology, Division of Oral Biological Sciences, Faculty of Dental Science, Kyushu University, 3-1-1 Maidashi, Higashi-ku, Fukuoka 812-8582, Japan, Email: taka-kanematsu@dent.kyushu-u.ac.jp


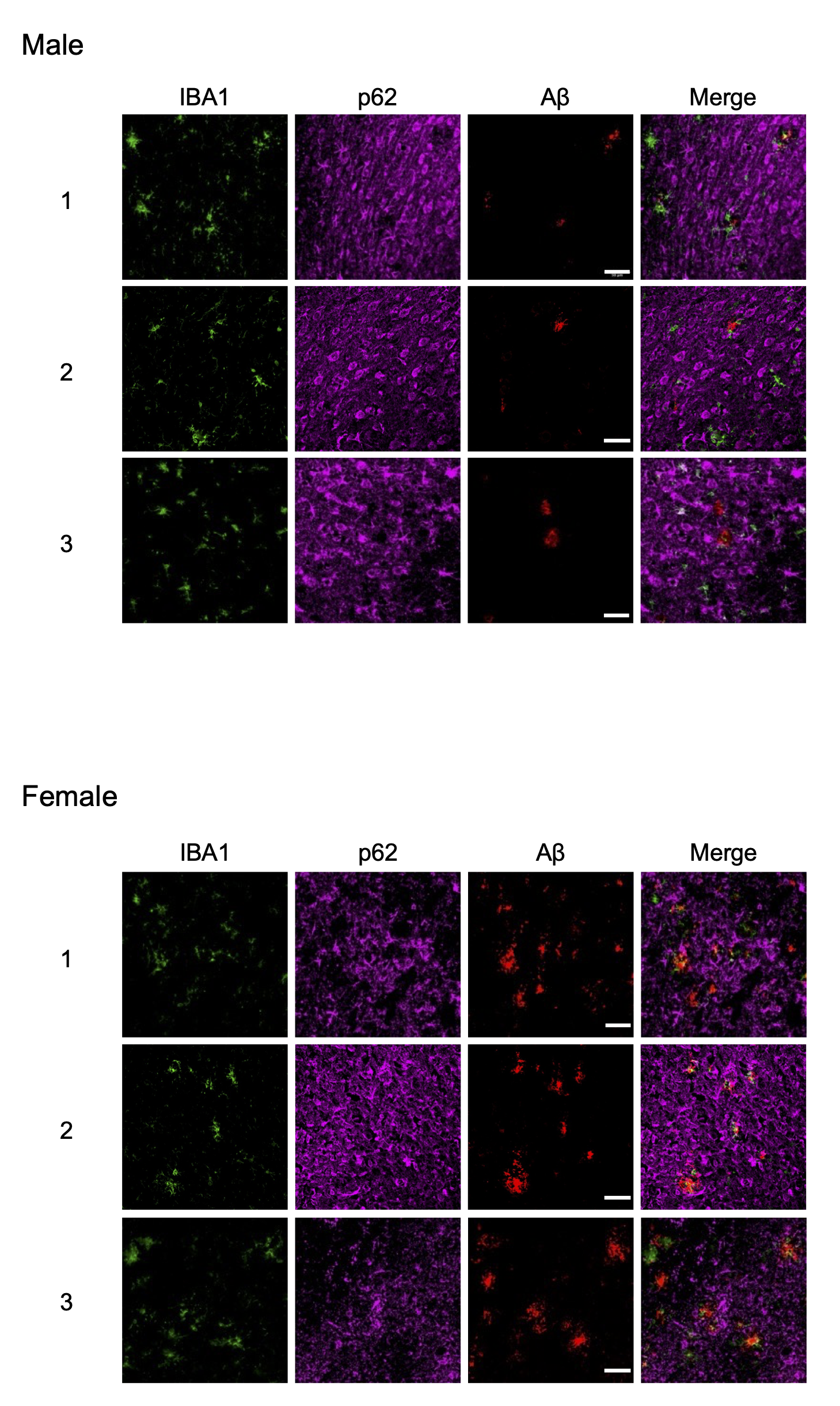


**Figure S1. Confocal images of the cerebral cortex of 5xFAD mice.** Additional representative images of cerebral cortex sections from 7-month-old male and female 5xFAD mice as in Figure 1 stained with anti-IBA1 (green), anti-p62 (magenta), and anti-Aβ (6E10, red) antibodies. Scale bar: 50 μm. Immunohistochemistry was performed on brain sections from two independent experiments per male or female, with 25 field views per section. Three sets of representatives from 50 images are shown.

**Figure S2. Western blotting images of testosterone-treated microglia. (A,B)** MG6 cells were treated with Aβ_1–42_ fibrils for indicated times. Bafilomycin A1 (+, 100 nM) or DMSO (−) was added for the final 2.5 h. Western blotting was performed using whole-cell lysates with anti-LC3B and anti-β-actin antibodies. The detected full images area shown in (A), and the optical density quantification of LC3-II relative to b-actin in the presence and the absence of bafilomycin A1 are shown in (B). Experiments were repeated five times. **p < 0.01 for the indicated comparison by one-way ANOVA followed by Tukey-Kramer’s HSD test. (**C–F**) MG6 (C, D) and HMC3 (E, F) cells were treated with Aβ_1–42_ fibrils for 24 h, followed by the addition of testosterone (+, 100 nM) or vehicle (−) for the final 2 h. Bafilomycin A1 (+, 100 nM) or DMSO (−) was added 30 min before testosterone treatment. Western blotting was performed using whole-cell lysates with anti-LC3B and anti-β-actin antibodies. The detected full images of the text Fig. 2A and 2B and the cropped area represented by a dotted square are shown in (C) and (E), respectively. Four (D) or two (F) additional image sets are shown. Arrowheads represent each detected band. Molecular weight size (kDa) for protein markers is shown in numbers. (**G**) Additional representative immunofluorescence images of MG6 cells stimulated with FITC-Aβ_1–42_ as in Figure 2D and E. The top row shows LC3-positive puncta in grayscale, and the middle row shows Aβ-positive areas, also in grayscale. The bottom row presents the merged images, with LC3 in red, FITC-Aβ in green, and nuclei in blue. Magnified images are shown below the merged images. Scale bars, 20 μm. (**H**) Full membrane images and other images of Figure 2E. MG6 cells were stimulated with Aβ_1–42_ fibrils for the indicated times in the presence and the absence of testosterone. The expression of p62 was determined in whole-cell lysates. The cropped areas shown as a dotted square in C, E, and H are used for the text images.


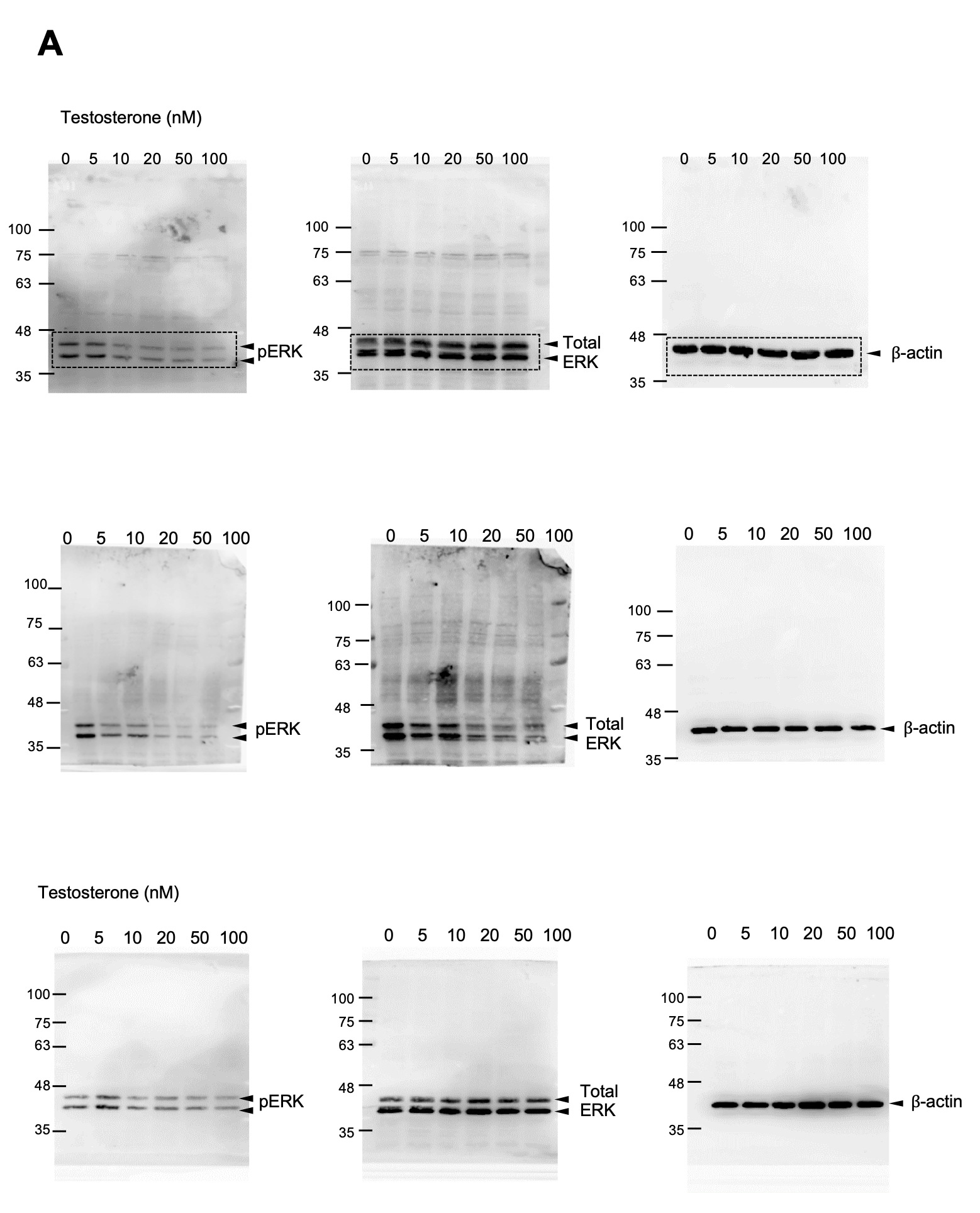

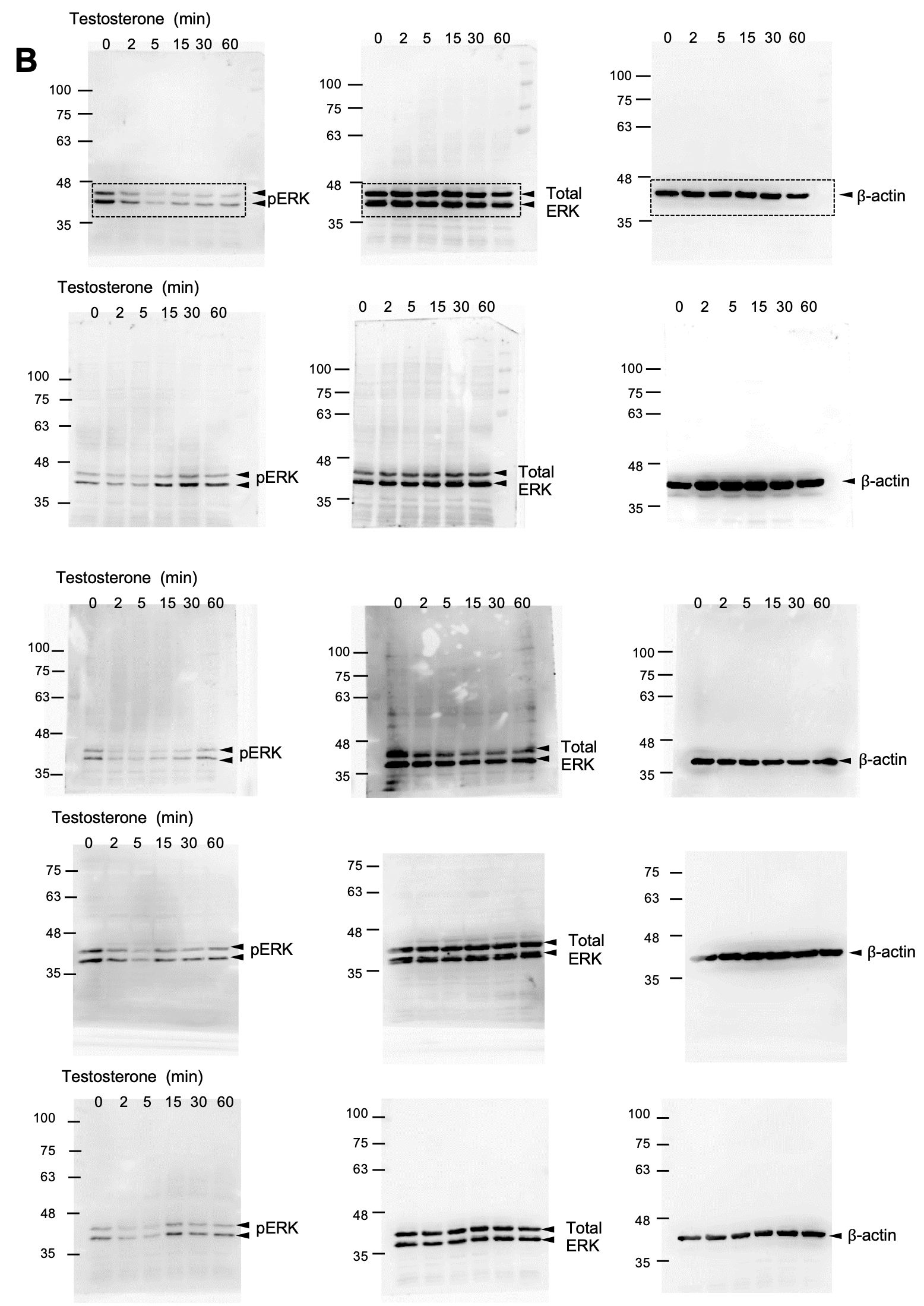

**Figure S3. Western blotting images of the expression of phosphorylated and total AKT after testosterone stimulation in MG6 cells and full membrane western blotting images for Figure 3.** (**A**) Western blotting analysis of total and phosphorylated forms of ERK in MG6 cells stimulated with testosterone for 1 h at different concentrations as in Figure 3B. (**B**) Western blotting analysis of total and phosphorylated forms of ERK in MG6 cells stimulated with 20 nM of testosterone for the indicated times as in Figure 3C. **(C, D)** MG6 cells were treated with 20 nM testosterone for indicated times. The expression of phosphorylated AKT at S473 (C) and T308 (D) along with total AKT were determined in whole-cell lysates. (**E**) GPRC6A was silenced in MG6 cells using shRNAs #1 and #2 and treated with 100 nM testosterone for 2 h. Full membrane images of the western blot for GPRC6A, phosphorylated ERK, total ERK, and β-actin are shown. The cropped areas shown as a dotted square in A, B, and E are used for the text images.


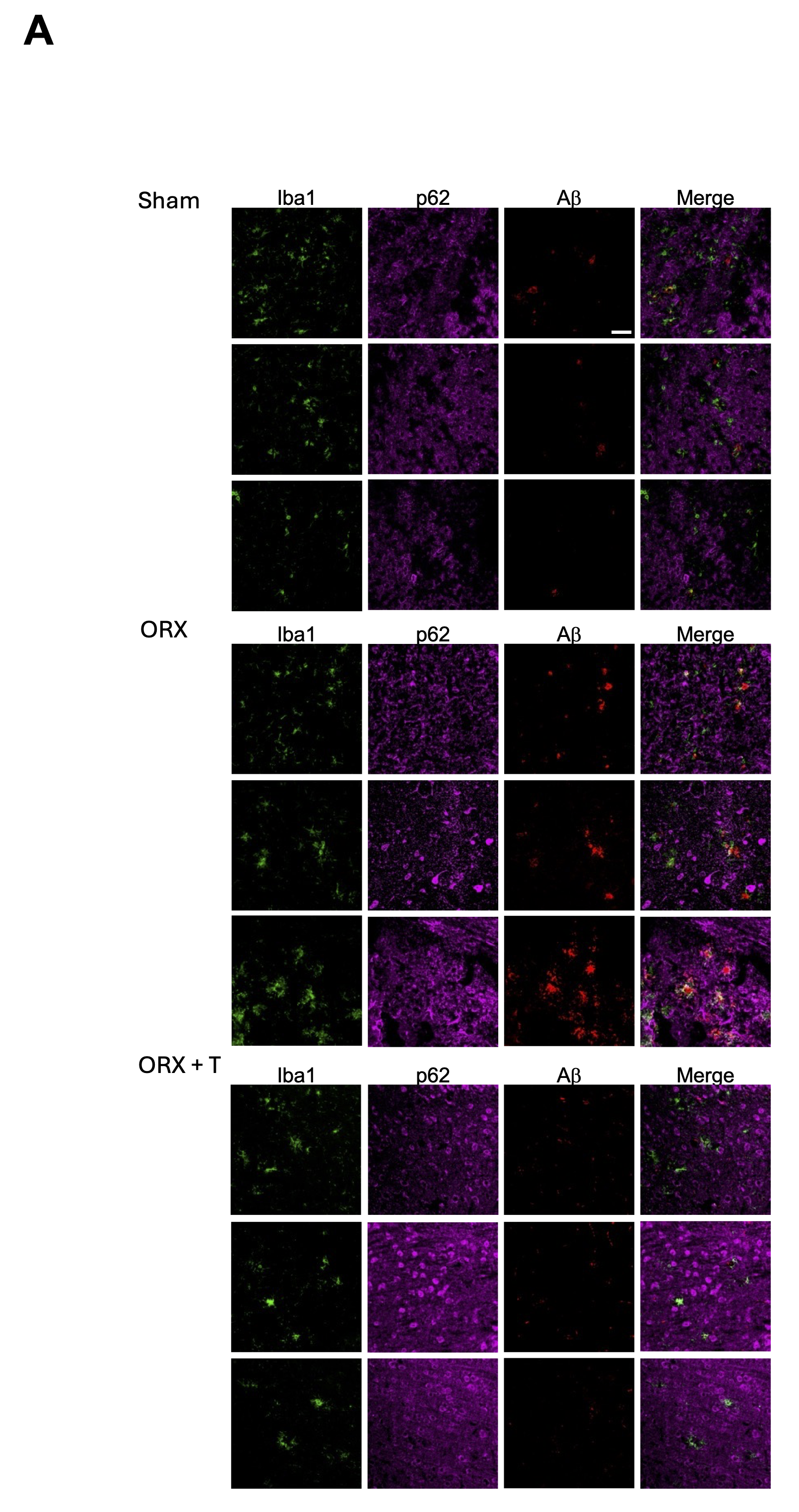

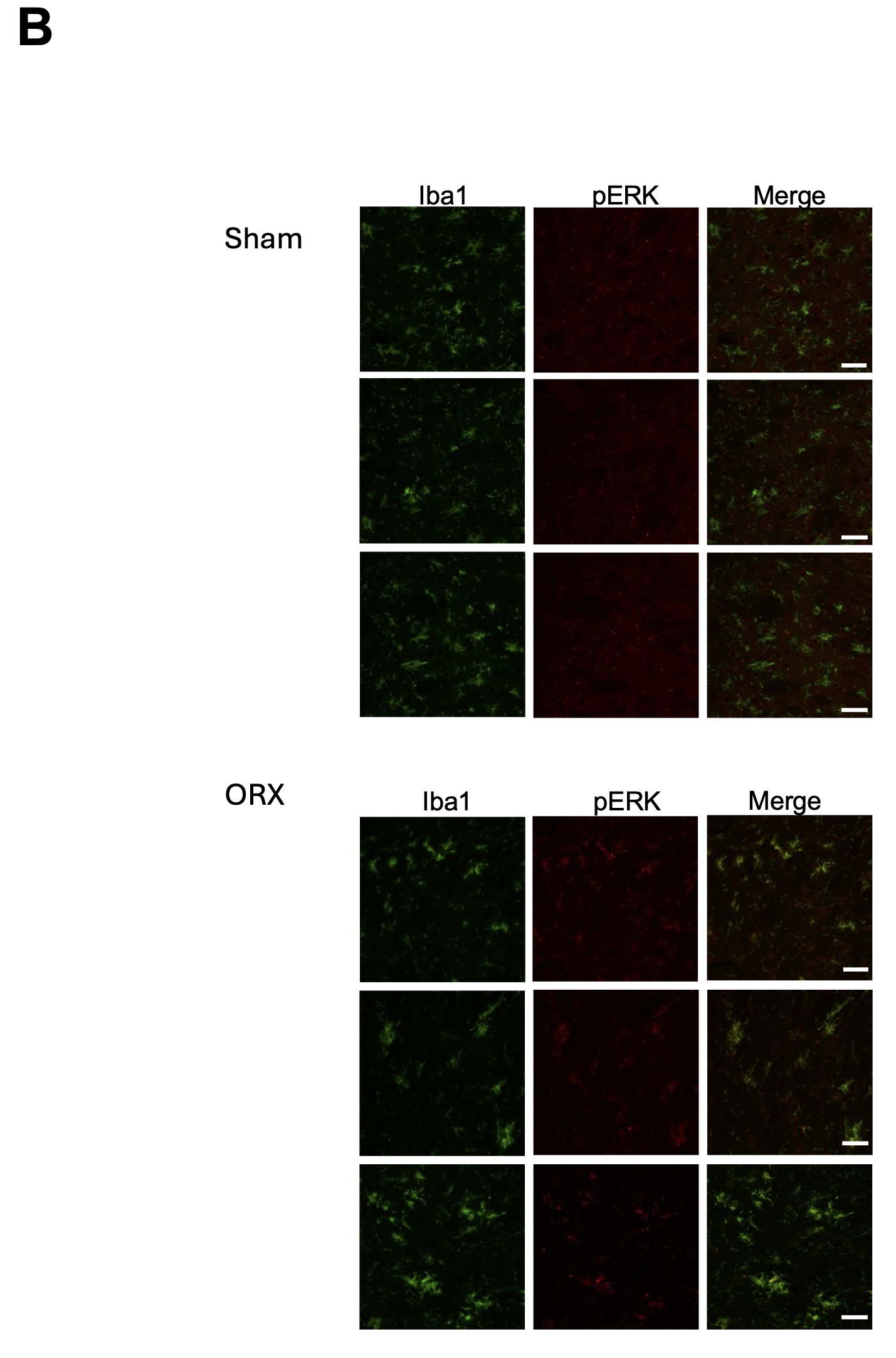


**Figure S4. Testosterone deprivation by testicular removal increases** **Aβ in the brain of 6-month-old 5xFAD mice.** (**A**) Additional representative images of cerebral cortex sections from 7-month-old male and female 5xFAD mice as in Figure 4A. (**B**) Additional representative images of cerebral cortex sections from 7-month-old male and female 5xFAD mice as in Figure 4D.


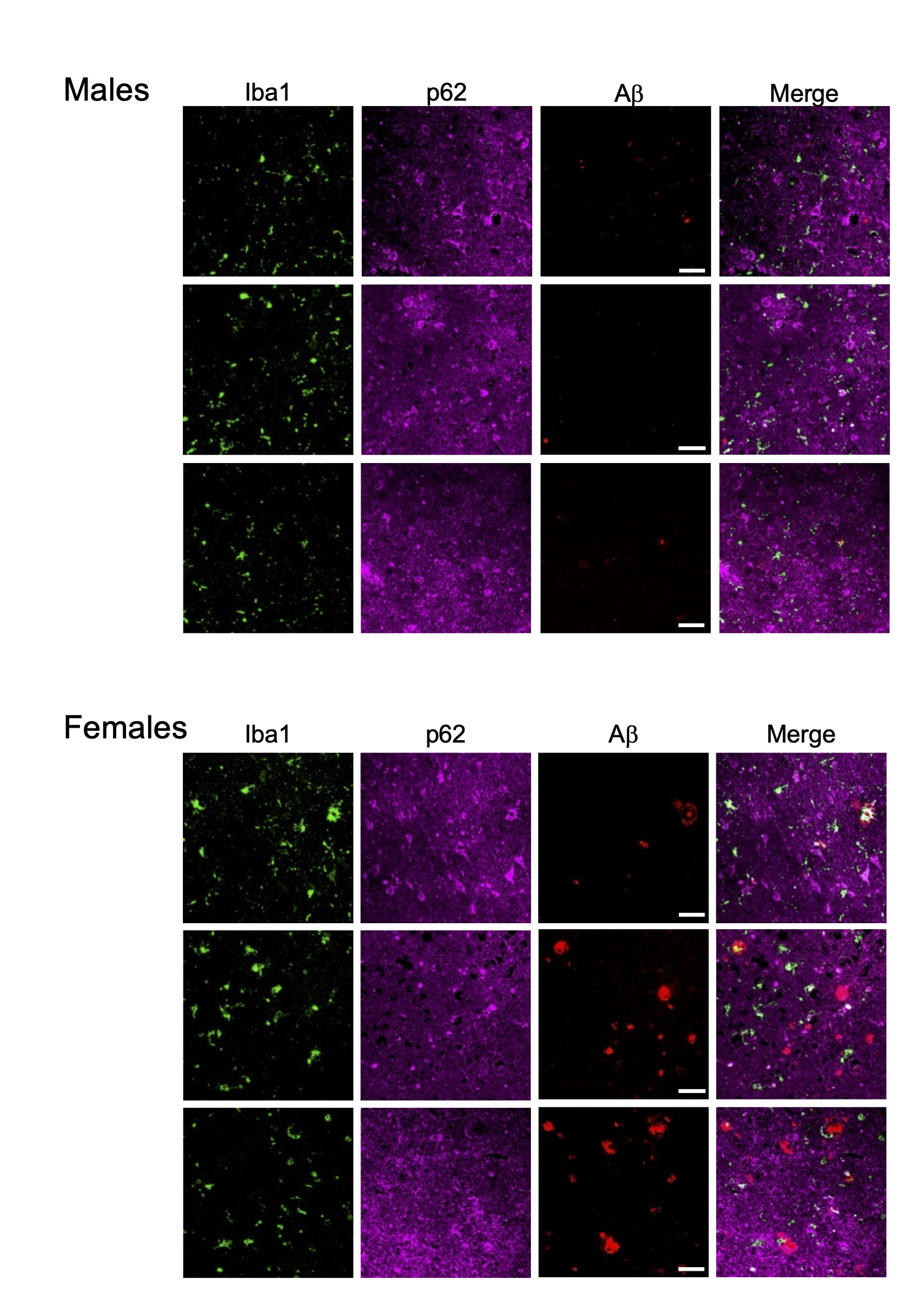


**Figure S5. Histological evaluation of microglial autophagy in plaque-associated microglial cells in the cortex of male and female patients with AD.** Additional confocal images of IBA1 (green), p62 (magenta), and Aβ (red) in the cortex of male and female patients with AD as shown in Figure 5. Scale bar: 100 μm.

**Table S1. Antibodies used for immunocytochemistry and western blot analysis**

| Head 1 [units]^a)^ | Head 2 | Head 3 |
| --- | --- | --- |
| Phospho-p44/42 MAPK (Erk1/2) (Thr202/Tyr204) (D13.14.4E) | 4370 | Cell Signaling Technology |
| Phospho-Akt (Ser473) (D9E) | Column 2 | Cell Signaling Technology |
| amyloid β (D54D2) | 8243 | Cell Signaling Technology |
| LC3B (E5Q2K) | 83506 | Cell Signaling Technology |
| LC3B (D11) | 3868 | Cell Signaling Technology |
| p70 S6K | GTX107562 | Gene Tex |
| Phospho-p70 S6k (T389) | 9234 | Cell Signaling Technologyv |
| Iba1 (EPR16589) | ab178847 | Abcam |
| β-Amyloid 1-16 APP (6E10) | SIG39320 | Bio Legend |
| LC3B | 14600-1-AP | Proteintech |
| p62/SQSTM1 | PM045 | MBL |
| SQSTM1/p62 | A11483 | ABclonal |
| GPRC6A | SAB2109176 | Sigma-Aldrich |
| β-actin (AC-15) | A1978 | Sigma-Aldrich |
| Anti-rabbit HRP | 7074 | Cell Signaling Technology |
| Anti-mouse HRP | 7076 | Cell Signaling Technology |
| Goat anti-Rabbit IgG (H+L) Alexa Fluor 488 | A11008 | Thermo Fisher Scientific |
| Goat anti-Rabbit IgG (H+L) Alexa Fluor 594 | A11012 | Thermo Fisher Scientific |
| Donkey Anti-Rabbit IgG H&L (Alexa Fluor 647) | ab150075 | Abcam |
| Donkey Anti-Mouse IgG H&L (Alexa Fluor 488) | ab150105 | Abcam |
| Donkey Anti-Mouse IgG H&L (Alexa Fluor 555) | ab150106 | Abcam |
| Donkey Anti-Rat IgG H&L (Alexa Fluor 488) | Ab150153 | Abcam |
